# Superoxide Release by Macrophages through NADPH Oxidase Activation Dominating Chemistry by Isoprene Secondary Organic Aerosols and Quinones to Cause Oxidative Damage on Membranes

**DOI:** 10.1021/acs.est.2c03987

**Published:** 2022-11-17

**Authors:** Ting Fang, Yu-Kai Huang, Jinlai Wei, Jessica E. Monterrosa Mena, Pascale S. J. Lakey, Michael T. Kleinman, Michelle A. Digman, Manabu Shiraiwa

**Affiliations:** † Department of Chemistry, University of California, Irvine 92697, California, United States; ‡ Department of Biomedical Engineering, University of California, Irvine 92697, California, United States; § Division of Occupational and Environmental Medicine, University of California, Irvine 92697, California, United States

**Keywords:** lipid peroxidation, cell membrane fluidity, cellular mechanism, antioxidant response elements, reactive oxygen species, aqueous chemistry, epithelial lining fluid

## Abstract

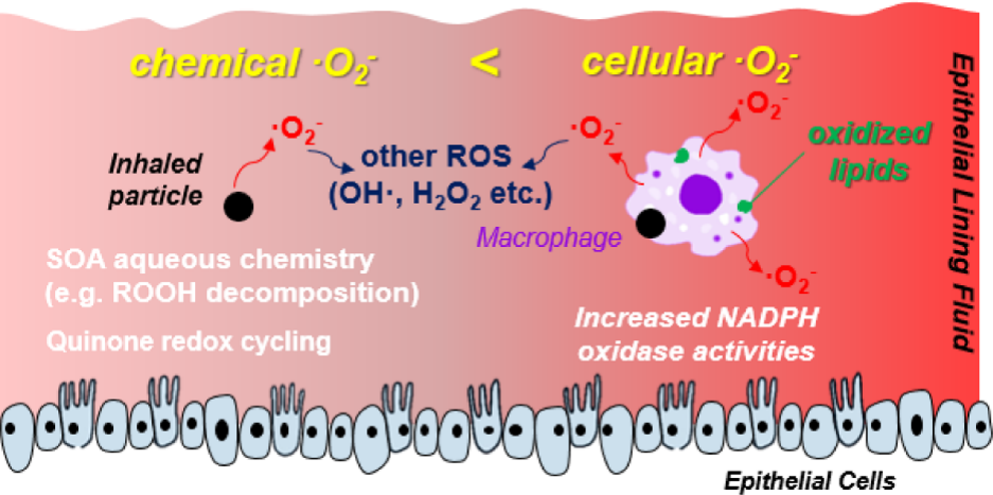

Oxidative stress mediated by reactive oxygen species (ROS) is a key process for adverse aerosol health effects. Secondary organic aerosols (SOA) account for a major fraction of fine particulate matter, and their inhalation and deposition into the respiratory tract causes the formation of ROS by chemical and cellular processes, but their relative contributions are hardly quantified and their link to oxidative stress remains uncertain. Here, we quantified cellular and chemical superoxide generation by 9,10-phenanthrenequinone (PQN) and isoprene SOA using a chemiluminescence assay combined with electron paramagnetic resonance spectroscopy as well as kinetic modeling. We also applied cellular imaging techniques to study the cellular mechanism of superoxide release and oxidative damage on cell membranes. We show that PQN and isoprene SOA activate NADPH oxidase in macrophages to release massive amounts of superoxide, overwhelming the superoxide formation by aqueous chemical reactions in the epithelial lining fluid. The activation dose for PQN is 2 orders of magnitude lower than that of isoprene SOA, suggesting that quinones are more toxic. While higher exposures trigger cellular antioxidant response elements, the released ROS induce oxidative damage to the cell membrane through lipid peroxidation. Such mechanistic and quantitative understandings provide a basis for further elucidation of adverse health effects and oxidative stress by fine particulate matter.

## Abstract 2

This study provides mechanistic and quantitative understandings of chemical and cellular superoxide release and oxidative stress induced by secondary organic aerosols.

## Introduction

Air pollution with high concentrations of fine particulate matter (PM) causes several millions of premature deaths per year globally.^1,2^ PM exposure is linked to oxidative stress and inflammation through the generation of reactive oxygen species (ROS) including superoxide (^•^O_2_^–^), the hydroxyl radical (OH^•^), and hydrogen peroxide (H_2_O_2_).^3^ ROS play a central role in physiological processes as signaling molecules,^4^ but excess generation of ROS can overwhelm the antioxidant defense capacity to cause oxidative stress and respiratory diseases.^5−7^ Among all ROS, ^•^O_2_^–^ is particularly of great interest as it can be converted by chemical and enzymatic processes into H_2_O_2_, which is a precursor of highly reactive OH^•^ radicals.^8−10^ A major fraction of fine PM is secondary organic aerosols (SOA),^11^ which are generated *via* atmospheric oxidation of volatile organic compounds (VOCs), forming a myriad of oxygenated compounds such as hydroperoxides and alcohols.^12,13^ In the aqueous phase, these compounds can undergo a cascade of chemical reactions to form ^•^O_2_^–^.^14^ Fine PM also contains redox-active compounds with high oxidative potential as introduced into the atmosphere from various sources including traffic-related emissions and biomass burning.^15^ Upon inhalation and respiratory deposition of PM in the epithelial lining fluid covering human airways, these compounds can trigger redox reactions to convert O_2_ into ^•^O_2_^–^.^16,17^ Hence, there are a growing number of oxidative potential measurements using acellular assays such as dithiothreitol and ascorbic acid assays emerging as new plausible metrics for PM toxicity.^15,18^

Macrophages are the first cellular responders of the innate immune system that protect the lung from infection through bacteria, microbes, and pathogens by releasing ^•^O_2_^–^ after phagocytosis through a process called the “respiratory burst” due to transient consumption of oxygen.^19^ This process also occurs upon exposure to atmospheric PM.^20−22^ Thus, ^•^O_2_^–^ can be generated both chemically and cellularly upon PM respiratory deposition; however, the relative importance of these pathways is poorly quantified. A previous study has quantified chemical and cellular H_2_O_2_ production from macrophages upon exposure to naphthalene SOA,^23^ but very limited research has been conducted in quantifying cellular ^•^O_2_^–^. Cellular release of ^•^O_2_^–^ can be triggered by a number of different enzymatic systems such as mitochondrial oxidative phosphorylation, NAD(P)H [reduced nicotinamide adenine dinucleotide (phosphate)] oxidase, and xanthine oxidase.^24^ However, very little is known about which ^•^O_2_^–^ generation mechanism is activated by inhaled PM. While ^•^O_2_^–^ can be converted into a less reactive form (*e.g.*, H_2_O_2_) by SOD, excess ^•^O_2_^–^ is known to be cytotoxic as it can interfere with lipids, proteins, and DNA,^6,7,19,25^ but a specific consequence of PM-triggered ^•^O_2_^–^ is yet to be identified. Several methods have been developed to detect cellular ^•^O_2_^–^ using spectroscopic,^26^ fluorescence,^27,28^ or luminescence^29,30^ assays. The Diogenes chemiluminescence assay is suitable for monitoring cellular ^•^O_2_^–^ production as Diogenes is a very sensitive ^•^O_2_^–^ chemiluminescence enhancer that is non-denaturing to living cells; however, the chemiluminescence readouts represent light intensity without providing a quantitative measure for the production rate or concentration.^30^

Here, we develop a method to quantify superoxide generation by combining the Diogenes chemiluminescence assay with electron paramagnetic resonance (EPR) spectroscopy. RAW 264.7 macrophages, a widely-applied and established macrophage cell line for studying the oxidative stress responses of macrophages^19,31−34^ (see also Table S1), are exposed to 9,10-phenanthrenequinone (PQN) and isoprene SOA. PQN is one of the most abundant quinones in atmospheric PM with cytotoxic effects both *in vitro* and *in vivo*.^35^ Quinones are important components in anthropogenic SOA as they are generated by the oxidation of polycyclic aromatic hydrocarbons and they can also be directly emitted *via* diesel exhaust, tire wear, and biomass burning associated with soot and humic-like substances.^36,37^ Quinones are redox active and accept electrons from antioxidants to form semiquinones,^38,39^ which can react with O_2_ to form ^•^O_2_^–^, which can further be converted into H_2_O_2_.^8^ This pathway is a major source of H_2_O_2_ for naphthalene SOA.^40^ Isoprene is the most abundant biogenic VOC emitted from plants. The isoprene SOA represents a major component of biogenic PM,^12,13^ and it has been found to induce oxidative stress in human lung cells.^41^ Quinones and isoprene SOA can also generate superoxide by chemical processes, which we quantify using EPR with a spin-trapping technique and kinetic modeling. The phasor approach to fluorescence lifetime imaging (Phasor-FLIM) is a state-of-the-art cellular imaging technique that has been previously used to study cellular metabolism in detail.^42−45^ Here, we apply Phasor-FLIM for the first time to study NADPH oxidase activities as a potential mechanism of cellular ^•^O_2_^–^ release after PM exposure. In addition, we apply Laurdan FLIM and the third harmonic generation (THG) microscopy^46^ to investigate the impacts of PQN and isoprene SOA on cell membrane fluidity and lipids, respectively.

## Methods

### Sample Collection and Preparation

Isoprene SOA particles were generated with ^•^OH photo-oxidation in a 19 L potential aerosol mass (PAM) chamber^47^ (see Figure S1 for the schematic of the system). 100–500 μL of isoprene was placed in an open 1.5 mL amber glass vial, which was kept inside a glass bottle. VOC gases were mixed with a carrier flow of 0.5 L min^–1^ of purified air from a zero-air generator (model 7000, Environics) and combined with humidified zero air (Perma Pure humidifier, MH-110-12P-4) prior to the PAM chamber inlet. ^•^OH was generated by UV radiation (185 nm) through the photolysis of water molecules with a relative humidity of ∼40%. The flow rate in the chamber was 6.5 L min^–1^, resulting in a mean residence time of approximately 3 min. While ^•^OH concentrations are higher (∼10^10^ cm^–3^) than ambient levels (∼10^6^ cm^–3^), the PAM-generated SOA are found to be similar to ambient and chamber-generated SOA in terms of the yield, oxidation state, hygroscopicity, and chemical composition.^48−50^ Multiple PAM experiments were conducted, and the isoprene SOA had a mean particle diameter of 400 nm with a mass concentration of particles of ∼100–1000 μg m^–3^, as measured using a scanning mobility particle sizer (SMPS, Grimm Aerosol Technik). The SOA particles were collected on pre-weighed 47 mm polytetrafluoroethylene (PTFE) filters (Millipore FGLP04700, 0.2 μm pore size) at a flow rate of 5 L min^–1^ for 6 h. The filter samples were weighed and stored at −18 °C for less than a week before analysis. The SOA samples were extracted (with an extraction efficiency of 86 ± 3%) and diluted in different volumes of the incomplete medium (Dulbecco’s modified Eagle medium, DMEM, GIBCO) to achieve various doses for cell exposure. A 5 mM PQN stock solution was prepared in dimethyl sulfoxide and kept in a freezer (−18 °C). The PQN solutions and SOA extracts were diluted in incomplete medium and kept at room temperature (roughly 25 °C) for no longer than 2 h before cell exposure.

### Cell Culture and Cytotoxicity

Macrophage cells (ATCC TIB-71) were obtained and passaged in complete medium (DMEM supplemented with 10% FBS and 1% penicillin streptomycin) until >80% confluent. Cells were then seeded at a density of 4 × 10^4^ cells/mL with 200 μL per well into 96-well plates (Corning) and incubated at 37 °C and 5% CO_2_ in an incubator for about 2 h for cells to fully adhere to the bottom of the culture plate. Cell density was kept the same for superoxide measurements and cell imaging and is within the typical ranges used for PM exposure studies in other studies (details in Table S1).

Cell cytotoxicity was measured using the CellTox Green Cytotoxicity Assay, which measures the changes in membrane integrity that may occur as a result of cell death. In brief, after cells were cultured in 96-well plates, the complete medium was replaced by 100 μL of incomplete medium and 50 μL of the CellTox Green reagent for each well. The CellTox Green reagent was produced according to the package directions. To facilitate dye/DNA binding, cells were incubated in the dark for at least 15 min before 50 μL of samples was added to initiate exposure. Fluorescence signals were then measured using a microplate reader (Promega, GloMax) for 4 h with excitation and emission wavelengths of 475 and 525 nm, respectively. The fluorescence signals were given as the relative light unit (RLU).

### Superoxide Measurements

A Diogenes chemiluminescence assay combined with EPR spectroscopy was applied to quantify cellular and chemical ^•^O_2_^–^ production with and without superoxide dismutase (SOD) pretreatments. The Diogenes probe reacts with ^•^O_2_^–^ to emit flash a chemiluminescence signal that is proportional to the ^•^O_2_^–^ production rate. The output of the Diogenes chemiluminescence is in the RLU. To convert the RLU to ^•^O_2_^–^ production rates, the Diogenes chemiluminescence assay was calibrated with EPR coupled with a spin-probe technique using the standardized cell-free ^•^O_2_^–^ generation system—the hypoxanthine (HX) and xanthine oxidase (XO) system (Figure S2). ^•^O_2_^–^ production rates calculated from the EPR spin probe method show a linear relationship with the RLU from chemiluminescence (*R*^2^ = 0.993, Figure S3). Therefore, the slope from the linear regression was used to convert the Diogenes chemiluminescence data to the ^•^O_2_^–^ production rate in the unit of micromoles per minute (see further details in the Supporting Information).

**Figure 1:**
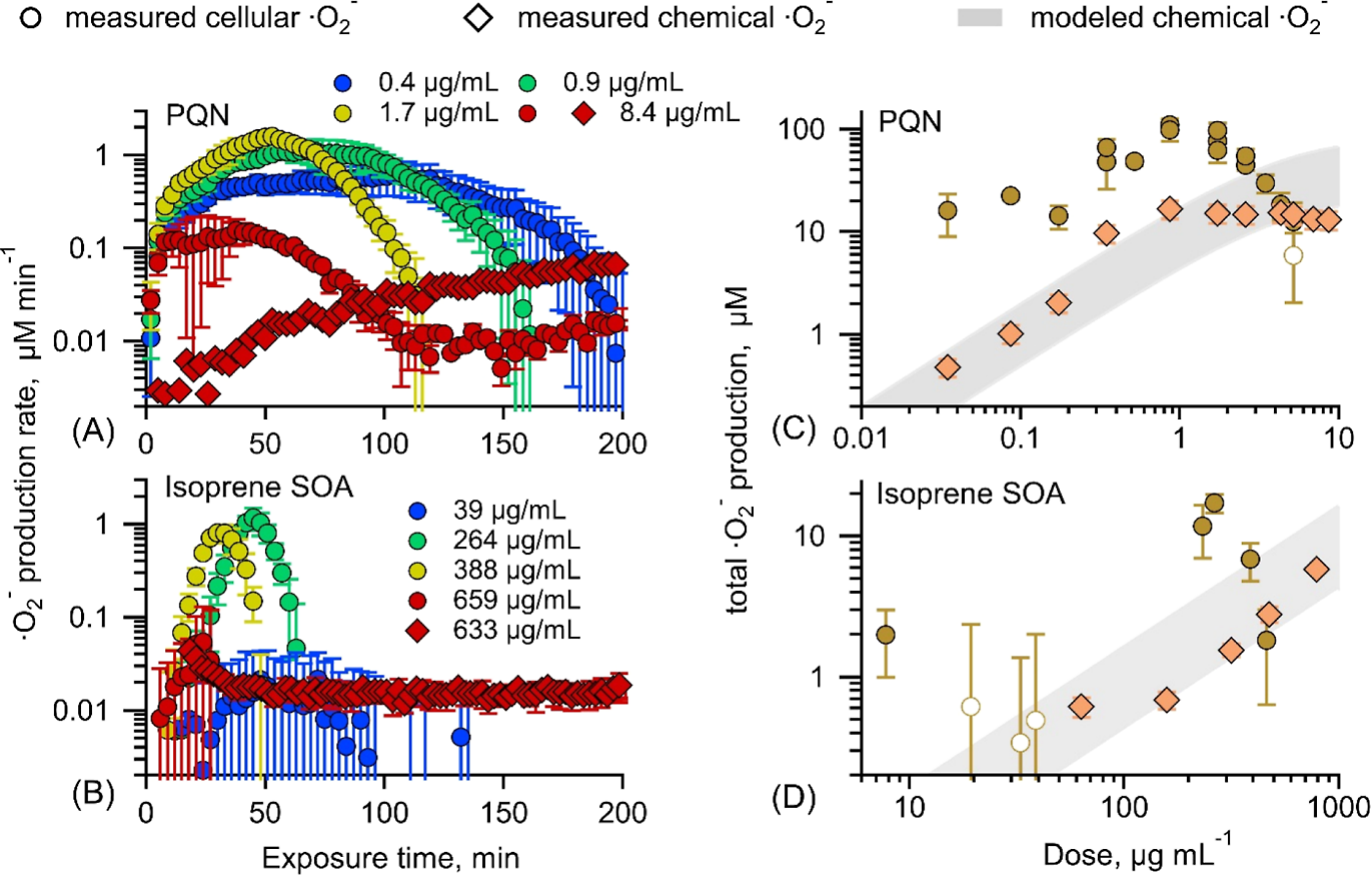
Time profiles (A, B) and dose–response curves (C, D) of cellular (circles) and chemical (diamonds) ^•^O_2_^-^ production upon exposure to PQN and the isoprene SOA. Markers in panels (A) and (B) are color-coded and labeled with the doses (in μg mL^–1^). Data points with error bars represent the average and uncertainties calculated from error propagation based on variabilities from samples and controls (see the Statistical Analyses section for details). Exposure groups statistically insignificant (p-value > 0.05, unpaired *t*-test) compared to vehicle controls are plotted as open circles in C and D. Chemical production of ^•^O_2_^-^ was also simulated using kinetic models with shaded areas representing model uncertainties.

For cellular ^•^O_2_^–^ measurements, after the cells were cultured in 96-well plates, the medium was gently removed, and 50 μL of the Diogenes probe made in sterile water was added to each well to incubate for 2 min. Then, 100 μL of incomplete medium (for SOD-pretreated wells, 85 μL of incomplete medium, and 15 μL of 1250 U mL^–1^ SOD in PBS buffer were added instead), and 50 μL of the sample was added to start the exposure. For inhibitor experiments that used Apocynin (Apo) as a specific NADPH oxidase inhibitor,^51^ 10 μL of Apo (roughly 100 μM) was added before sample exposure. In order to prevent cross-well interferences, black sealing films (AbsorbMax, EXCEL Scientific, Inc.) were used to seal the bottom of the plates. Continuous luminescence measurements were conducted using the plate reader every 3 min for 4 h to obtain the ^•^O_2_^–^ production rate (Figure 1A,B). The total ^•^O_2_^–^ production (Figure 1C,D) was obtained by integrating the area under the curve from the kinetics of ^•^O_2_^–^ release as a function of exposure time. Incomplete medium and filter blanks were used as vehicle controls for PQN and SOA, respectively. The ^•^O_2_^–^ production rate and total production from exposure to samples were corrected by subtracting those from controls so that the control-corrected data represent cellular ^•^O_2_^–^ induced by exposure to samples. Unexposed (*i.e.*, vehicle controls) macrophages release ^•^O_2_^–^ at ∼0.06 μM min^–1^ with a total production of ∼14 μM (Figure S4), which represents ^•^O_2_^–^ production from a normal metabolism regulated by mitochondrial respiration.^6^ Cell-free wells were also used as “acellular controls” to capture any background signals, which were subtracted from the data with cells when necessary.

The measurements of chemical ^•^O_2_^–^ production followed the same protocol as that of cellular ^•^O_2_^–^ measurements except cells were absent and the reaction time started when PQN or isoprene SOA particles were dissolved or extracted in incomplete medium (pH 7.4). Accounting for the time it took to prepare samples, chemical ^•^O_2_^–^ measurements usually started at 15 min.

### Kinetic Modeling

A kinetic multi-layer model of the surface and bulk chemistry in the epithelial lining fluid (KM-SUB-ELF)^16^ was used to estimate the chemical ^•^O_2_^–^ production from PQN. KM-SUB-ELF treats mass transport and chemical reactions involving ROS, antioxidants, surfactants, and redox-active compounds in the epithelial lining fluid. The model considers the redox cycling reactions between ascorbate and quinones to generate ^•^O_2_^–^.^4,25^ The model simulations were conducted with PQN doses ranging from 0 to 10 μg mL^–1^. Initial ascorbate concentrations of 10 and 40 μM^52^ were used to obtain total ^•^O_2_^–^ production with a reaction time of 4 h. The model results from 10 and 40 μM ascorbate correspond to the upper and lower bounds of the KM-SUB-ELF model in Figure 1C.

Chemical ^•^O_2_^–^ production from the isoprene SOA was estimated using a SOA aqueous kinetic model from our previous work.^14^ The kinetic model includes chemical reactions of SOA components, ROS coupling reactions, and radical–radical reactions. SOA chemistry includes decomposition of organic hydroperoxides (ROOH), generating ^•^OH, ^•^OH oxidation of primary and secondary alcohols (ROH), and the subsequent reaction with O_2_ to form α-hydroxyperoxyl radicals, which can decompose to generate the hydroperoxyl radical HO_2_^•^, and ^•^OH oxidation of SOA components. The model simulations were conducted with initial SOA concentrations ranging from 0 to 1000 μg mL^–1^, 50% of ROH, and 1–4% ROOH of total SOA mass. Model uncertainties are associated with uncertainties in the relative abundance of ROOH and ROH groups in the SOA.

### Phasor Approach to Fluorescence Lifetime Imaging

The NADPH oxidase complex is an important source of ^•^O_2_^–^ in phagocytosis as activated by bacterial products and cytokines.^53^ The activation of NADPH oxidase generates ^•^O_2_^–^ through the reaction: NADPH + 2O_2_ → NADP^+^ + H^+^ + 2^•^O_2_^–^.^54,55^ Here, we utilize the auto-fluorescence of NADPH measured using FLIM combined with experiments using NADPH oxidase activator PMA^56^ and inhibitor Apocynin to study the NADPH oxidase activities. It should be noted that NADH (nicotinamide adenine dinucleotide) has identical fluorescence properties to NADPH,^57^ and thus, FLIM cannot differentiate between NADH and NADPH, and we denote the FLIM signals as NAD(P)H. Note that NADH reacts with oxygen to produce either water or H_2_O_2_^58^ or produces significantly lower ^•^O_2_^–^ than NADPH.^59^ NAD(P)H expresses in two forms inside cells, bound and free states. Each state has its own fluorescence lifetime, and in the Phasor-FLIM method, the NAD(P)H fluorescence lifetime is visualized in polar coordinates by calculating the Fourier sine and cosine transformations of the FLIM photon histogram curve.^43^ Since oxidized NAD(P)^+^ does not emit auto-fluorescence, the conversion of NAD(P)H to NAD(P)^+^ reduces the fraction of bound NAD(P)H and causes a shift from the bound end to free end of the NAD(P)H free-bound trajectory.^60,61^ Therefore, the relative locations on the trajectory can be used to obtain the bound NAD(P)H fractions, and the decrease of bound NAD(P)H fractions indicates the activation of NADPH oxidase activities (see further details in the Supporting Information).

**Figure 2:**
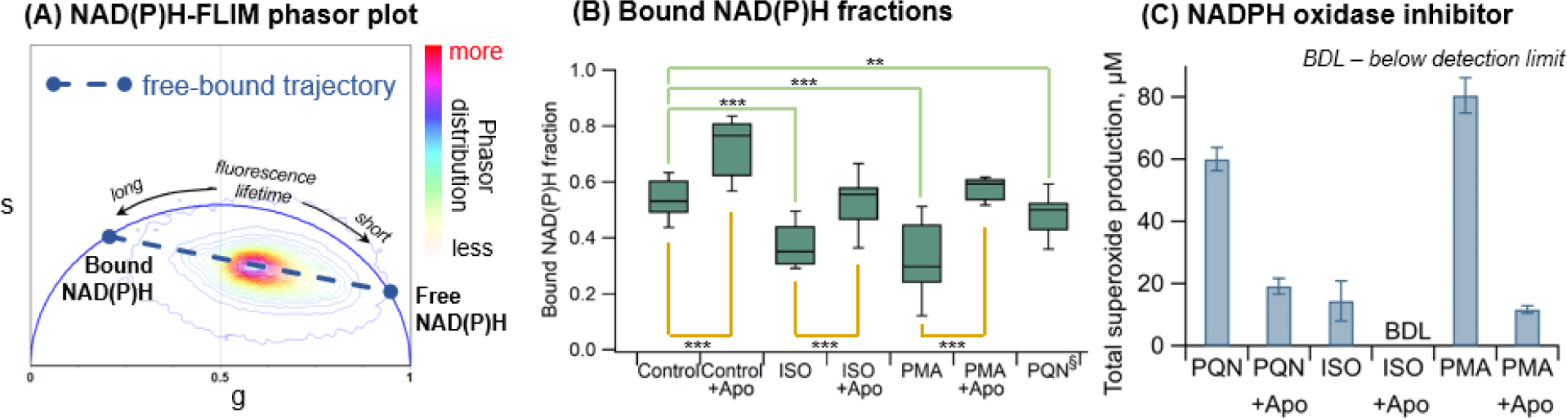
NADPH oxidase activities. (A) Phasor plot of the FLIM images from RAW 264.7 macrophages treated with the control (vehicle) and all samples. (B) NAD(P)H bound fractions based on phasor locations in (A) for macrophages treated with the control and samples with and without NADPH oxidase inhibitor apocynin (Apo). (C) Effect of apocynin on total ^•^O_2_^-^ production. Bars with error bars represent the average from triplicates and the standard deviation. Unpaired *t*-test, ***p < 0.0001 and **p < 0.001. ISO and PMA denote isoprene SOA and phorbol 12-myristate 13-acetate, respectively. ^§^FLIM images were taken from cells after 10 min exposure of PQN, washed with PBS buffer, and replaced with fresh incomplete medium.

For FLIM imaging, cells were seeded at a density of 4 × 10^4^ cells mL^–1^ and incubated overnight before exposure to PQN and isoprene SOA for imaging. PQN and SOA in media in the absence of cells produced low fluorescence background signals. Cells were imaged in a 37 °C and 5% CO_2_ environment before exposure and at different time points after the addition of samples (see Table S2). Since NAD(P)H mainly resides in cell membranes and cytoplasm^62^ and since the fluorescence lifetimes of nucleus do not change significantly before and after sample exposure (Figure S5), the nucleus was cropped out, that is, only the fluorescence signals from the cell membranes and cytoplasm were included in calculating the bound NAD(P)H fractions. The bound NAD(P)H fractions from all data points for each sample were averaged from multiple cells (*N* = 12–23) and are shown in Figure 2B. PQN exhibited an unusually long fluorescence lifetime distribution, and the phasors from PQN fall outside of the NAD(P)H free-bound trajectory. We conducted hyperspectral imaging to confirm that the long fluorescence lifetime was caused by phosphorescence from triplets of PQN (see the Supporting Information and Figure S6).^63^ To avoid the lifetime of phosphorescence interfering with the NAD(P)H fluorescence lifetime, we used PBS buffer to wash cells after 10 min of incubation. After replacing with fresh incomplete medium, cells were loaded back to the FLIM system for imaging. Using this method, the lifetime phasors of PQN fall onto the NAD(P)H free-bound trajectory, as shown in Figure S5.

The FLIM Laurdan imaging technique was used to measure cell membrane fluidity as a result of sample exposure.^64^ Laurdan (6-dodecanoyl-2-dimethylaminonaphthalene) is a fluorescent membrane marker used to investigate membrane fluidity. Shorter Laurdan fluorescence lifetimes correspond to increases in membrane fluidity due to lipid peroxidation. After seeding cells in an eight-well plate, complete medium was replaced with incomplete medium, and Laurdan dye was added to each well to achieve a final concentration of 5 μM and incubated under the cellular condition for 30 min before exposure to the control, PQN, PMA, and isoprene SOA. After some period of exposure, the cells (4 × 10^4^ cells mL^–1^) were loaded to the FLIM system for imaging at 800 nm excitation wavelength (see further details in the Supporting Information and Table S2).

### Third Harmonic Generation Imaging

The THG imaging technique was applied to detect the accumulation of lipids inside the cells after exposure to isoprene SOA and PQN.^46^ The excitation of the THG signal requires laser wavelengths of up to ∼1000 nm, which conventional tunable Titanium Sapphire lasers could not achieve. The THG imaging was carried out in the deep imaging via emission recovery (DIVER) system from the LFD, which uses Spectra Physics Insight DS + femtosecond laser tunable in the range of 68–1300 nm. The actual focal depth difference was found to be ∼0.75 mm for different wavelength excitations, which for NAD(P)H is 740 nm and for THG is 1050 nm. The THG signal is generated at the interface between media with the difference in the third-order nonlinear susceptibility, refractive index, and dispersion and can be used to detect lipids. The THG images were taken before and after 10 min exposure to isoprene SOA and PQN. Note that THG microscopy is not affected by long-lifetime phosphorescence from PQN.

### Statistical Analyses

For Diogenes chemiluminescence data, for each dose, triplicate measurements were performed on the same experimental day along with vehicle and acellular controls, which were used to correct the experimental data. Experiments with some different doses were performed on different days. One-way ANOVA followed by Tukey’s post-hoc test on the total superoxide production from the same dose of PQN (1.74 μg mL^–1^) performed on different days shows that no significant difference was determined between the mean of data (*p* > 0.1). The time profile of superoxide production and total production are presented as the mean of the triplicate measurements with error bars representing the uncertainties propagated from the standard deviations of triplicate measurements of samples and controls. For imaging, all data points for each sample were averaged from multiple cells (*N* > 10). Results were analyzed by using unpaired student *t*-tests. The results were considered statistically significant at a of *p*-value ≤ 0.05 for all exposure groups compared to control groups.

## Results and Discussion

### Dose–Response Relationship of Superoxide Production

Figure 1A,B shows the measured temporal evolution of control-corrected chemical and cellular ^•^O_2_^–^ production rates upon exposure to selected doses of PQN and isoprene SOA, respectively. Note that similar temporal trends are observed in other doses. The cellular ^•^O_2_^–^ production rate increases to ∼0.4 μM min^–1^ when exposed to 0.4 μg mL^–1^ PQN. Upon an increase of the dose to 0.9 and 1.7 μg mL^–1^, cellular ^•^O_2_^–^ production rates increase and reach higher maxima at ∼1 and ∼1.6 μM min^–1^ after ∼50 min, respectively, followed by decreases over time. At a higher dose of 8.4 μg mL^–1^, macrophages respond faster but reach a lower peak at ∼0.2 μM min^–1^ at 40 min and decrease more swiftly afterward. At all doses, cellular ^•^O_2_^–^ production rates eventually decrease to the basal level, indicating the completion of cellular response after exposure to samples. In the absence of cells, PQN with a dose of 8.4 μg mL^–1^ triggers redox reactions of semiquinone that can react with O_2_ to produce ^•^O_2_^–^. The reduction of PQN to its semiquinone form is the limiting step, leading to a gradual increase of O_2_^–^ production rate to reach ∼0.1 μM min^–1^ over a few hours.

Similar temporal trends are observed for cellular ^•^O_2_^–^ release from the isoprene SOA (Figure 1B), while much higher doses are required to trigger ^•^O_2_^–^ release from macrophages. Upon increasing doses from 39 to 659 μg mL^–1^ of the isoprene SOA, cellular ^•^O_2_^–^ production rates reach their peaks above 1 μM min^–1^ for 264 μg mL^–1^ and ∼0.05 μM min^–1^ for 659 μg mL^–1^. While PQN stimulates macrophages for ^•^O_2_^–^ release for a few hours, the isoprene SOA induces it for less than 2 h. In the absence of cells, 633 μg mL^–1^ of the isoprene SOA produces ^•^O_2_^–^*via* aqueous reactions of SOA components with a comparable rate to the peak cellular release rate. Compared to the cellular release, the chemical ^•^O_2_^–^ production rate from the isoprene SOA stays relatively constant (Figures 1B and S7) due to the relatively fast onset of aqueous reactions. Note that a previous study by Tong *et al.*(40) did not observe the formation of chemical ^•^O_2_^–^ by the isoprene SOA in the surrogate lung fluid (pH of 7.4) using the EPR spin trapping technique. We have very recently found that the EPR spin trap method is not ideal to measure ^•^O_2_^–^ in neutral conditions due to the low trapping rate of ^•^O_2_^–^,^65^ which likely explains the difference between Tong *et al.* and this work.

The dose–response relationships are shown in Figure 1C,D, where responses are given as total ^•^O_2_^–^ production as obtained from the integration of control-corrected ^•^O_2_^–^ production rates over exposure time. The measured chemical total ^•^O_2_^–^ production increases with an increase of dose, and it plateaus at ∼10 μM. PQN generates ^•^O_2_^–^*via* redox-cycling reactions with antioxidants,^4,16^ while the isoprene SOA yields ^•^O_2_^–^*via* a series of aqueous reactions including decomposition of α-hydroxyperoxyl radicals and organic hydroperoxides as well as OH oxidation of primary or secondary alcohols.^14^ We applied kinetic models that include these chemical mechanisms to simulate chemical ^•^O_2_^–^ production,^14,16^ producing consistent results as the measurements within model uncertainties (shaded areas). While the model does not perfectly reproduce the observed temporal evolution of the ^•^O_2_^–^ production rate (Figure S7), this agreement is still remarkable, given that the reaction system is very complicated with numerous reactions involved;^14^ the discrepancies may be due to incomplete chemical mechanisms, untreated species contained in media, and the inexplicit treatment of pH that can modulate ROS formation.^66^

Interestingly, cellular ^•^O_2_^–^ release induced by PQN and the isoprene SOA shows inverted U shape dose–response distributions. Under low doses, macrophages release ^•^O_2_^–^ under regular cellular metabolisms with a control level of ∼14 μM (Figure S4B), which is higher than the chemical ^•^O_2_^–^ production (<0.05 μM). Once the dose reaches a threshold, macrophages are activated, and cellular total ^•^O_2_^–^ production increases sharply, further dominating over chemical ^•^O_2_^–^ production. The onset dose of cell activation to release an excess amount of ^•^O_2_^–^ for PQN (∼0.2 μg mL^–1^) is much lower than for the isoprene SOA (>40 μg mL^–1^), indicating that macrophages are more sensitive to PQN than to the isoprene SOA in producing ROS. This suggests that quinones are intrinsically more toxic than the isoprene SOA, consistent with previous studies that show that anthropogenic aromatics have higher toxicity compared to biogenic aerosols.^67−69^

Cellular ^•^O_2_^–^ production induced by PQN and isoprene SOA then decreases at higher doses, surpassed by chemical ^•^O_2_^–^ production (PQN at ∼5 μg mL^–1^ and SOA at ∼450 μg mL^–1^). The decreases of cellular ^•^O_2_^–^ release at higher doses (Figure 1C,D) and longer exposure time (Figure 1A,B) are not due to cell death. Cell cytotoxicity measurements show that cells exposed to the doses of up to 5 μg mL^–1^ for PQN and 923 μg mL^–1^ for the isoprene SOA have similar fluorescence signals (unpaired *t*-test, *p*-value > 0.05) to that of filter blanks (fresh filter without particle collection), which were much lower than those from the positive control lysed cells (*p*-value < 0.05) (Figure S8), confirming that cells are alive during the whole course of exposure. Instead, the decreased cellular release of ^•^O_2_^–^ is most likely due to redox homeostasis by cells upregulating the antioxidant response elements to scavenge ROS for protection against proinflammatory effects.^70^ Previous studies have found that antioxidant enzymes can be upregulated by the transcription factor Nrf2 when oxidative stress is induced upon exposure to ambient and diesel exhaust PM containing polycyclic aromatic hydrocarbons and quinones.^20,71,72^ Based on the kinetics of cellular ^•^O_2_^–^ releases (Figure 1A,B), the differences in the rates of decrease and time to reach peak production rates from different doses suggest that the activation of antioxidant response elements is also dose-dependent. The inverted U shape time profiles and the dose–response relationship also imply that the cellular antioxidant defense mechanisms occur after the activation of macrophage to release ^•^O_2_^–^.

The conversion of doses to ambient PM concentrations is highly complex as respiratory deposition is largely affected by the nature of breathing (nasal *vs* oral, tidal volume, and frequency), individual differences in the lung anatomy, the airflow patterns in the lung airways, and the presence of deposition hot spots in lung.^73^ Phalen *et al.* (2006) demonstrated by dosimetry model predictions that the surface PM deposition can reach up to 85.5 μg cm^–2^ but vary by up to 3 orders of magnitude in the tracheobronchial region.^74^ With a bottom area of 0.32 cm^2^ and a working volume of 0.2 mL of a 96-well plate, the surface doses for the SOA and PQN in the current study are 5–412 and 0.02–5.3 μg cm^–2^, respectively. Combining all the uncertainties and variabilities (up to 4 orders of magnitude) and also considering that the surface area of the alveolar region is about 200 times larger and the deposition in the alveolar region is up to 3 folds higher than in the tracheobronchial region,^75^ the applied surface doses are within relevant ranges for real-life exposure scenarios and consistent with doses applied in previous submerged cell exposure studies (details in Table S1).

### Cellular Superoxide Release by NADPH Oxidase Activation

We then applied cellular imaging techniques to study the mechanism of cellular ^•^O_2_^–^ release. Selected doses that activate massive cellular ^•^O_2_^–^ release (with minor contribution from chemical ^•^O_2_^–^ formation) were used for exposure (Table S2). The phasors for cell membranes and cytoplasm mainly fall along a metabolic trajectory represented by a line joining positions of free NAD(P)H and protein-bound NAD(P)H on a phasor plot (Figure 2A).^43,45^ The relative locations on the trajectory were used to obtain the bound NAD(P)H fractions (Figure 2B). Macrophages exposed to PQN and the isoprene SOA show lower bound NAD(P)H fractions than the controls, indicating that the bound-state NADPH is oxidized to non-fluorescent NADP^+^, releasing superoxide: NADPH + 2O_2_ → NADP^+^ + H^+^ + 2^•^O_2_^–^.^54,55^ A bound-to-free shift of NAD(P)H is observed upon exposure to phorbol 12-myristate 13-acetate (PMA), commonly used as an inducer to activate NADPH oxidase and increase endogenous ^•^O_2_^–^ production (Figure S9). Additionally, apocynin (or Apo), a specific NADPH oxidase inhibitor, results in higher bound NAD(P)H fractions (Figure 2B) in cells than those without inhibitors. Note that inhibitor experiments for PQN are not available due to the interference by phosphorescence of PQN. Taking into account the difference in the doses and exposure times for FLIM (Table S2), the bound fractions in Figure 2B for PQN and the isoprene SOA are not directly comparable. Consequently, apocynin reduces cellular ^•^O_2_^–^ production substantially upon exposure to PQN, the isoprene SOA, and PMA (Figure 2C). These observations strongly indicate that PQN and the isoprene SOA trigger cellular ^•^O_2_^–^ production mainly through activating the NADPH oxidase. We observe that the bound NAD(P)H fraction for controls with Apo is higher than isoprene SOA and PMA with Apo. The higher fraction in controls may be explained by the increase of NADH bound fractions from the increase of mitochondrial oxidative phosphorylation (OXPHOS), as a recent study showed that Apo can enhance ATP production and mitochondrial membrane potential, which are determined by the regulation of OXPHOS.^76^ This may further suggest that the exposure to PQN and the isoprene SOA induced a certain level of mitochondrial dysfunction, although future dedicated studies are necessary to further investigate this aspect.

### Oxidative Stress on Cell Membranes

**Figure 3:**
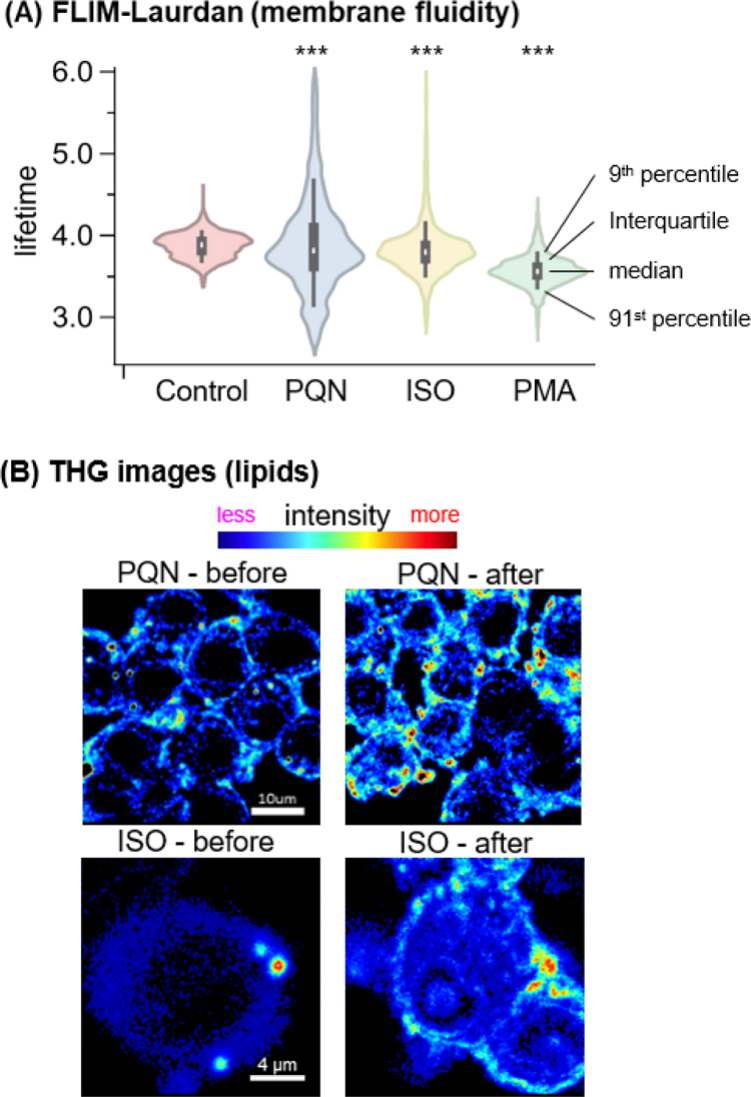
Oxidative stress on cell membranes. (A) Membrane fluidity measured by Laurdan FLIM. (B) Locations and intensity of lipid accumulation from THG imaging on cells treated with PQN (1.74 μg mL^–1^) and isoprene SOA (ISO, 305 μg mL^–1^) before and after 10 min exposure. Unpaired *t*-test, ***p < 0.0001.

To investigate the impact of PQN and the isoprene SOA on cell membranes, we quantified the lifetime changes of a solvatochromic probe (Laurdan) and the THG images of cells after exposure. The images of FLIM-Laurdan are shown in Figure S10. Since Laurdan dye is incorporated into the hydrophobic phase in the membrane,^64^ only the fluorescence signals from cell membranes were selected for fluorescence lifetime calculation. A total of 15 cells from each sample were averaged and are presented as violin plots in Figure 3A. We observe significant decreases in the fluorescence lifetimes of the Laurdan probe on cell membranes from macrophages exposed to PQN, isoprene SOA, and PMA, indicating that these samples cause an increase in membrane fluidity, an important parameter in membrane integrity and cell health. In addition, the THG microscopy imaging shows that exposure to PQN and the isoprene SOA causes an increase of the THG signal around cell membranes, suggesting the accumulation of lipids (Figure 3B). Bright-field cell images also suggest that macrophages may have taken up oxidized low-density lipoprotein (LDL), forming foam cells (Figure S11), a key event suggested to involve the activation of NADPH oxidase observed in previous studies.^77,78^ The doses for PQN and the isoprene SOA are 1.74 and 305 μg mL^–1^, respectively, and cellular ^•^O_2_^–^ dominates chemical production. Therefore, with the revelation of increased membrane fluidity and laden lipids around the cell membranes, we suggest that macrophages are undergoing lipid peroxidation caused by ^•^O_2_^–^ or other ROS produced thereafter. Note that the Diogenes probe can diffuse through the cell membrane, but SOD is membrane-impermeant. The addition of SOD largely eliminated the chemiluminescence intensity, suggesting that the observed cellular ^•^O_2_^–^ signals were primarily from extracellular ^•^O_2_^–^. This is consistent with NADPH oxidase being a transmembrane protein that regulates redox signaling by reducing extracellular O_2_ to ^•^O_2_^–^.^53^ This is also consistent with the observed oxidative stress induced by PQN and the isoprene SOA, which occurred mainly on the cell membrane by lipid peroxidation. Liu *et al.* (2022) suggested that the diffusion of chemically formed H_2_O_2_ into cells is a main contributor to intracellular H_2_O_2_ upon exposure to the naphthalene SOA.^23^ As the chemical lifetime of ^•^O_2_^–^ is much shorter than that of H_2_O_2_, ^•^O_2_^–^ is not expected to readily pass though the cell membranes. Although the penetration of ^•^O_2_^–^ through the membrane could still occur with the assistance of anion channels,^79^ our study indicates that this pathway is not significant. Note that the dismutation of extracellular ^•^O_2_^–^ can occur and the product H_2_O_2_ can diffuse across the membrane into the cytoplasm to cause oxidative damage intracellularly.

### Multi-Tier Response Mechanism

**Figure 4:**
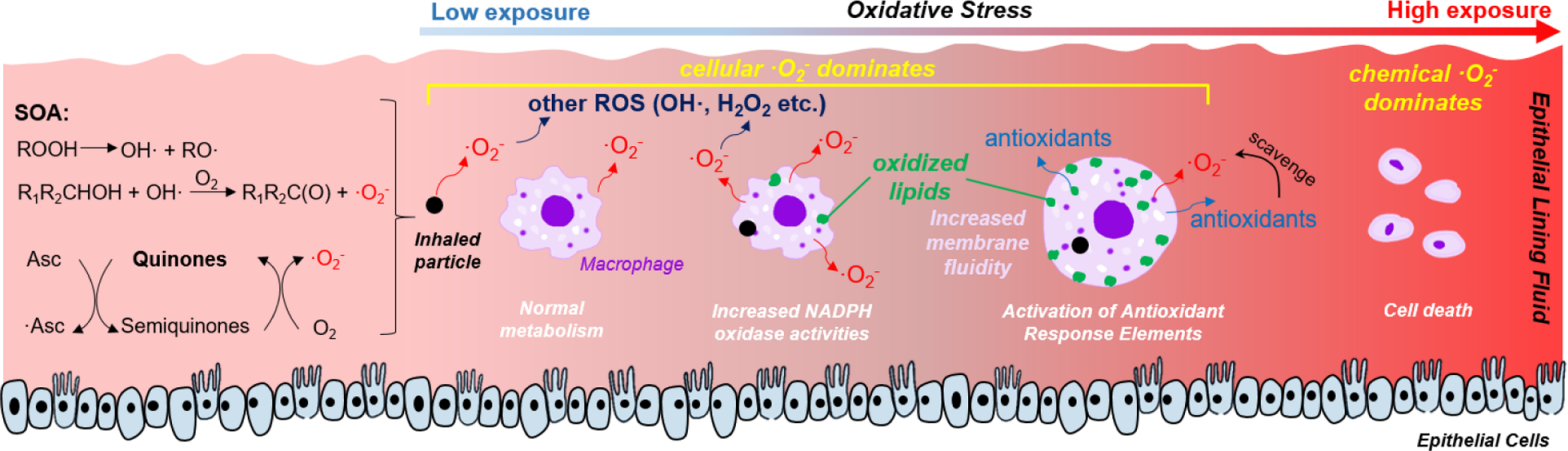
Multi-tier chemical and cellular response mechanisms upon PM deposition in the epithelial lining fluid. Cellular ^•^O_2_^-^ release via activation of NADPH oxidase mostly dominates over chemical ^•^O_2_^-^ production, causing lipid peroxidation and activation of antioxidant response elements.

Figure 4 summarizes multi-tier chemical and cellular response mechanisms upon PQN and SOA exposure in the epithelial lining fluid. At low doses, ^•^O_2_^–^ is mainly produced from normal cellular metabolism *via* mitochondrial respiration with minor contributions from chemical reactions. After a threshold dose to macrophages, NADPH oxidase activities are upregulated for respiratory burst, releasing massive amounts of ^•^O_2_^–^, which can cause oxidative stress by increasing cell membrane fluidity through lipid peroxidation. Further increases of doses lead to the activation of antioxidant response elements, reducing the net cellular ^•^O_2_^–^ production. At very high doses and long exposure times, chemical ^•^O_2_^–^ production becomes comparably important or dominant if the escalation of oxidative stress leads to cell death. Given that cellular ^•^O_2_^–^ release mostly dominates over its chemical production by PM reactive and redox-active components, widely-applied acellular assays that measure oxidative potential and ROS activity may need to be interpreted with caution.

The mechanistic understandings obtained in this study provide a basis for further elucidation of adverse health effects and oxidative stress upon respiratory deposition of PM. Future studies should be extended to include other chemical compounds that have high oxidative potential and redox activity such as transition metals; the presence of metals in ambient PM might enhance the importance of chemical ROS.^17,80−82^ While the current work is based on the standard submerged cell culture method, this classical condition should be extended to represent more realistic conditions with multiple types of cells including macrophages and epithelial and endothelial cells to simulate synthetic interactions between cell populations^83^ with an application of the air–liquid interface for simulating exposure and respiratory deposition of aerosol particles.^67^

## Supporting Information Available

Additional experimental details, materials, and methods, including chemicals and materials, SOA generation by the PAM chamber, cell culture, cytotoxicity, calibration of the Diogenes chemiluminescence assay and EPR spin probe, Phasor-FLIM, phosphorescence from PQN, and FLIM-Laurdan (PDF)
