# Supplementary material for: Superoxide Release by Macrophages through NADPH Oxidase Activation Dominating Chemistry by Isoprene Secondary Organic Aerosols and Quinones to Cause Oxidative Damage on Membranes
Source: Environ Sci Technol. 2022 Nov 17;56(23):17029–38. doi: 10.1021/acs.est.2c03987 (PMC9730850; doi:10.1021/acs.est.2c03987)
Supplement: Supplementary file 1 — es2c03987_si_001.pdf [file es2c03987_si_001.pdf]

Supporting Information

Superoxide Release by Macrophages through NADPH Oxidase Activation Dominating Chemistry by Isoprene Secondary Organic Aerosols and Quinones to Cause Oxidative Damage on Membranes

Ting Fang[1], Yu-Kai Huang[2], Jinlai Wei[1], Jessica E. Monterrosa Mena[3], Pascale S. J. Lakey[1], Michael T. Kleinman[3], Michelle A. Digman[2], Manabu Shiraiwa[1*]

[1]Department of Chemistry, University of California, Irvine, CA, USA, 92697.
[2]Department of Biomedical Engineering, University of California, Irvine, CA, USA, 92697.
[3]Division of Occupational and Environmental Medicine, University of California, Irvine, CA, USA, 92697.

*Correspondence to: m.shiraiwa@uci.edu.

Number of pages	15
Number of figures	11
Number of tables	2

Chemicals and materials

Diogenes Chemiluminescence Assay kits were obtained from National Diagnostics; CellTox™ Green Cytotoxicity Assay kits were obtained from Promega; 1-Hydroxy-3-methoxycarbonyl-2,2,5,5-tetramethylpyrrolidine (CMH) and 5-tert-Butoxycarbonyl-5-methyl-1-pyrroline-N-oxide (BMPO) (≥99%) were from Enzo Life Sciences; superoxide dismutase was from MP Biomedicals; toluene (99.7%) and Apocynin (Apo or 4′-Hydroxy-3′-methoxyacetophenone) were from Alfa Aesar; 9,10-Phenanthrenequinone (PQN), isoprene (≥ 99%), hypoxanthine (HX), and xanthine oxidase (XO) were purchased from Sigma-Aldrich; and Phorbol 12-myristate 13-acetate (PMA) was obtained from Fisher BioReagents.

Calibration of Diogenes chemiluminescence assay and EPR-spin probe

A spin probe CMH was used to react with $\cdot O_2^-$ to form EPR-detectable nitroxides radical CM· that has a characteristic three-peak EPR spectra.[1] CM· is stable over time and thus it can be used to calculate the $\cdot O_2^-$ production rate. Instrument parameters for EPR spectra acquisition were set to the following: a microwave frequency of 9.652 GHz; a modulation frequency of 100 kHz; a modulation amplitude of 1 G; a center field of 3520 G; a sweep width of 80 G; a microwave power of 20 mW; a number of scans of 5; a receiver gain of 30 dB. Concentrations of $\cdot O_2^-$ were obtained by simulating the CM· spectra and counting the total number of spins using the SpinFit and SpinCount modules embedded in the Bruker Xenon software.

With the catalysis of XO, HX is oxidized to xanthine which is then oxidized to uric acid. This oxidation process produces electrons that are passed to dioxygen to produce $\cdot O_2^-$.[2, 3] The concentration of HX was fixed at 250 μM and XO was varied in the range of 0-0.6 mU mL^{-1} (U as the enzyme unit in μmol min^{-1}). First, XO and probe reagent (either 50 μL Diogenes or 15 μL 3mM CMH) were mixed in incomplete media, yielding negligible chemiluminescence or CM· signal, shown as "background" in Figure S2. The slight increase of "background" signals were likely due to auto-oxidation of CMH.[4] Next, HX was added to initiate the reaction in a final volume of 200 μL. Chemiluminescence and EPR measurements were then carried out with 1.5 to 7 minutes of reaction. Figure S2 shows an example of CM· and chemiluminescence signals over reaction time. Pretreatments with SOD diminished the signals to the background levels, confirming that the enhanced signals were due to the presence of $\cdot O_2^-$.

$\cdot O_2^-$ production rates calculated from the EPR-spin probe method show a linear relationship with RLU from chemiluminescence ($R^2 = 0.993$, Fig. S3). Therefore, the slope from the linear

regression was used to convert the Diogenes chemiluminescence data to $\cdot O_2^-$ production rate in the unit of μM min^{-1}. Note that the small intercept from the regression was not taken into account when converting the Diogenes chemiluminescence data as it is the background CM· level due to CMH auto-oxidation in the EPR-CMH method.

Phasor-FLIM

FLIM measures the time a fluorophore spends during excited state before emitting a photon then returning to ground state which can vary from picoseconds to hundreds of nanoseconds. Lifetime measurements take advantage in absolute measurements independent from probe concentration or absorption, luminescence intensity. Furthermore, the phasor approach has a robust computation analysis and provides additional dimension to fluorescence data mapping. The phasor approach takes in fluorescence decay intensity from FLIM and Fourier-transforms either the multi- or mono-exponential lifetime into phasor plots on a g-s coordinate. The coordinates g and s represent the x- and y-axis, respectively, in a phasor plot. The conversion of fluorescence intensity to phasor plots are based on the following equations:

$$g_{i,j}(\omega) = \frac{\int_0^\infty I_{i,j}(t)\cos(\omega t)dt}{\int_0^\infty I_{i,j}(t)dt} \quad \text{(Eq. 1)}$$

$$s_{i,j}(\omega) = \frac{\int_0^\infty I_{i,j}(t)\sin(\omega t)dt}{\int_0^\infty I_{i,j}(t)dt} \quad \text{(Eq. 2)}$$

$$\omega = 2\pi f \quad \text{(Eq. 3)}$$

where $I_{ij}(t)$ is the fluorescence decay at each pixel; i and j indicate the pixel within the image; ω is the angular modulation frequency; and f is the laser repetition frequency. In our experiments, f is 80 MHz. Calibration of the system was done using 50 μM Coumarine 6 diluted in ethanol allowing determination of single-exponential lifetime of ~2.5 ns.

FLIM images were acquired with a Zeiss LSM710 META laser scanning microscope coupled to a 2-photon Ti:Saphirre laser (Mai Tai, Spectra Physics, Newport Beach, CA) for excitation at 740 nm using a 40x water-immersion objective (1.2 NA;Zeiss), and a photomultiplier tube (H7422P-40; Hamamatsu) for detection. An ISS A320 FastFLIM box was used to collect the fluorescence decay, and data were fourier transformed into g and s coordinates using the SimFCS software (Globals Software·G-SOFT LLC., Irvine UCI-CA) in the phasor approach. 20 frames of

images were collected for each time point. The bound NAD(P)H fraction of each macrophage cell can be obtained according to its relative location on the phasor plot.

Phosphorescence from PQN

PQN exhibited an unusually long lifetime distribution and relatively higher photon counts compared to other samples (over maximum 1 μs) in that the phasors from PQN fall outside of NAD(P)H free-bound trajectory, while those from nucleus remained on the trajectory (Fig. S4). This abnormal long lifetime was likely caused by delayed emission of phosphorescence from triplets of PQN.[5] The phosphorescence lifetime of aromatic ketones in a triplet state is known to be in the range of milliseconds for transitions forbidden by structure symmetry in the condition of low oxygen quenching. We verified the emission of phosphorescence from PQN using hyperspectral imaging (Fig. S5). Before exposure, cells' emission spectra exhibits a peak within a wavelength range of 450-525 nm, which is consistent with NAD(P)H emission wavelengths. After the same cells were exposed to PQN for 30 min, we observed significant increases in the intensity below 450 nm, likely due to the emission of phosphorescence from PQN. To avoid the lifetime of phosphorescence interfering with the NAD(P)H fluorescence lifetime, we used PBS buffer to wash cells after 10 minutes of incubation. After replacing with fresh incomplete media, cells were loaded back to the FLIM system for imaging. With this method, the lifetime phasors of PQN fall onto the NAD(P)H free-bound trajectory as shown in Fig. S4.

FLIM-Laurdan

The Laurdan (6-Dodecanoyl-2-Dimethylaminonaphthalene) is a solvatochromic probe that has the ability to attach in different depths and orientation of lipid bilayers and shift its spectral emission maximum upon hydrophobic environmental change. When Laurdan is in a hydrophobic environment, it has two excited states, the locally excited state, which is intrinsic to the fluorophore, and an internal charge transfer state created by a larger dipole moment. This polar relaxation causes water molecules in more hydrophilic environments to reorient to the same orientation of Laurdan's dipole that causes energy loss. According to former works done by the Laboratory of Fluorescence Dynamics (LFD), Laurdan lifetime in the blue channel decreases when the probe is in a more hydrophobic environment.[6] We measured the difference of Laurdan lifetime which does not require the need of fluorescence intensity. Lifetime imaging is relatively a more stable approach since it is independent on dye concentration and also provides local changes upon cell membrane. The dipolar relaxation effect of Laurdan is found to cause quenching of lifetime in both blue and

green channels, only that green channel does not shift along the phasor plot universal circle. Hence, we used a 460/80nm cubical filter to collect only blue emission spectra and project phasor points onto the universal circle to acquire absolute lifetime values.

Table S1. Cell exposure conditions from this and other studies

References	cell type	cell density, cells/mL	doses	exposure duration	sample
This study	RAW 264.7	4×10^4	0.035 – 8.5 µg/mL	4 h	9,10-phenanthrenequinone
This study	RAW 264.7	4×10^4	8 – 660 µg/mL	4 h	Isoprene-derived SOA
Wan et al. (2021)[7]	RAW 264.7	2×10^5	0-200 ng/mL	24 h	$PM_{2.5}$ extracts
Pardo et al. (2017)[8]	NR8383	6×10^4	833 µg/mL	6-24 h	Israel dust extracts
Pardo et al. (2015)[9]	fresh BAL cells	N/A	50 µg in whole lung	N/A	PM extracts
Tuet et al. (2016)[10]	MH-S & Primary ventricular myocytes	$(2–3)\times10^4$	8.3-83 µg/mL	24 h	$PM_{2.5}$ extracts
Wang et al. (2022)[11]	NR8383	2×10^5	<=25 µg/mL	2.5 h	$PM_{2.5}$ extracts
Zhang et al. (2008)[12]	NR8383	1×10^6	20-200 pg of PM per cell	2 h	$PM_{2.5}$ extracts
Gali et al. (2015)[13]	RAW 264.7	4×10^4	20-200 µg/mL	4 h	$PM_{2.5}$ extracts
Nishanth et al. (2011)[14]	RAW 264.7	4×10^5	5 µg/mL	24 h	Nanoparticles
Franzi et al. (2011)[15]	RAW 264.7	5×10^5	1 mg/mL	30 min-24 h	PM extracts
Xia et al. (2008)[16]	RAW 264.7	2.5×10^4	25 µg/mL	16-24 h	metal oxide nanoparticles & diesel exhaust particle extract
Imrich and Ning (1998)[17]	hamster AM & RAW 264.7	5×10^5	up to 400 µg/mL	30 min	residual oil fly ash
He et al. (2017)[18]	RAW 264.7	4×10^5	30 µg/mL	3-12 h	$PM_{2.5}$ extracts
Daher et al. (2011)[19]	NR8383	1×10^6	~167 µg/mL	2.5 h	PM extracts

Table S2. Doses of samples and exposure times at which FLIM images were taken. ISO denotes isoprene-derived SOA. NADPH oxidase inhibitor apocynin (Apo) is 100 µM/well. §Cells were washed with PBS after exposure to PQN for 10min.

Samples	Doses	NAD(P)H-FLIM		FLIM-Laurdan
		w/o. Apo	w. Apo	
Control	-	30-60 min	44-57 min	97-103 min
PQN	1.74 µg mL^{-1}	18-34 min§	-	70-79 min
ISO	~300 µg mL^{-1}	31-39 min	43-69 min	55-67 min
PMA	1 µM	20-32 min	36-41 min	33-50 min

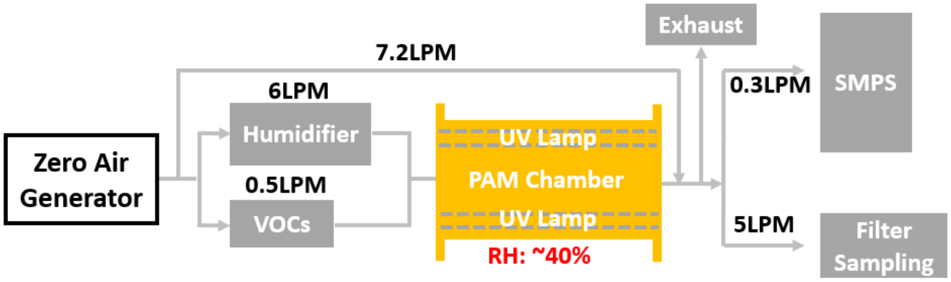

Figure S1. Schematic of SOA generation and the particle collection system.

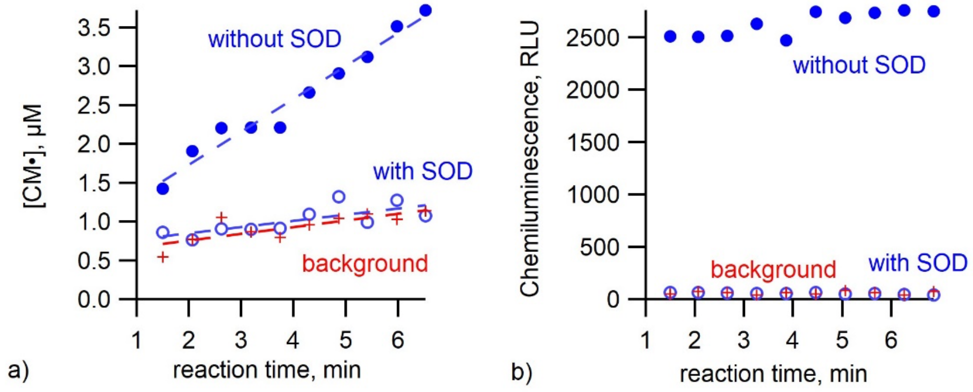

Figure S2. EPR and Diogenes chemiluminescence signal from the reaction between xanthine oxidase (XO) and hypoxanthine (HX) with and without the pretreatment of SOD. Background represents signals in absence of HX.

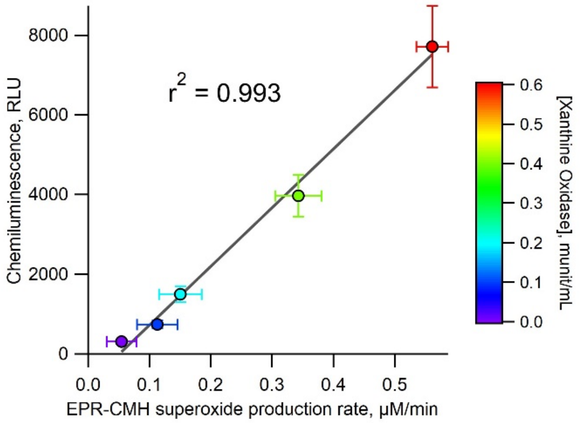

Figure S3. Relationship between Diogenes chemiluminescence and the $\cdot O_2^-$ production rate determined from the EPR-spin probe method. Data were expressed as the mean values of three independent experiments (N = 3).

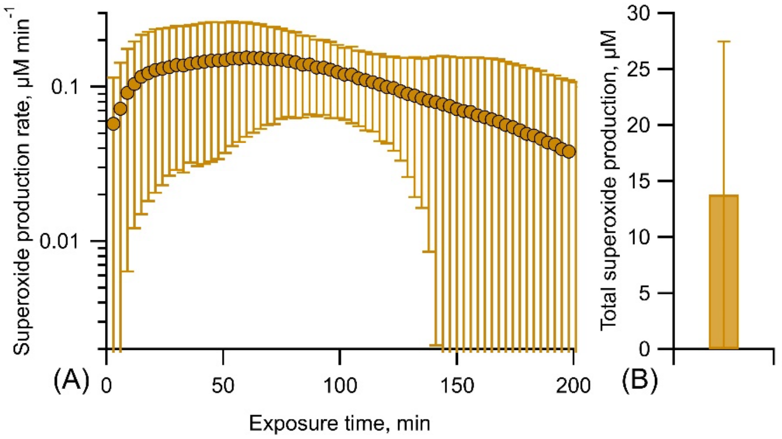

Figure S4. Time evolution of cellular $\cdot O_2^-$ production rates (A) and total basal production (B) from macrophages without sample exposure (vehicle controls, i.e., incomplete media and filter blanks extracted in incomplete media). Note the total basal production represents production from normal metabolism estimated from initial production rates assuming basal production do not significantly change for 4 hours. Data were averaged (± SD) from data obtained from control experiments conducted on different days (N = 20).

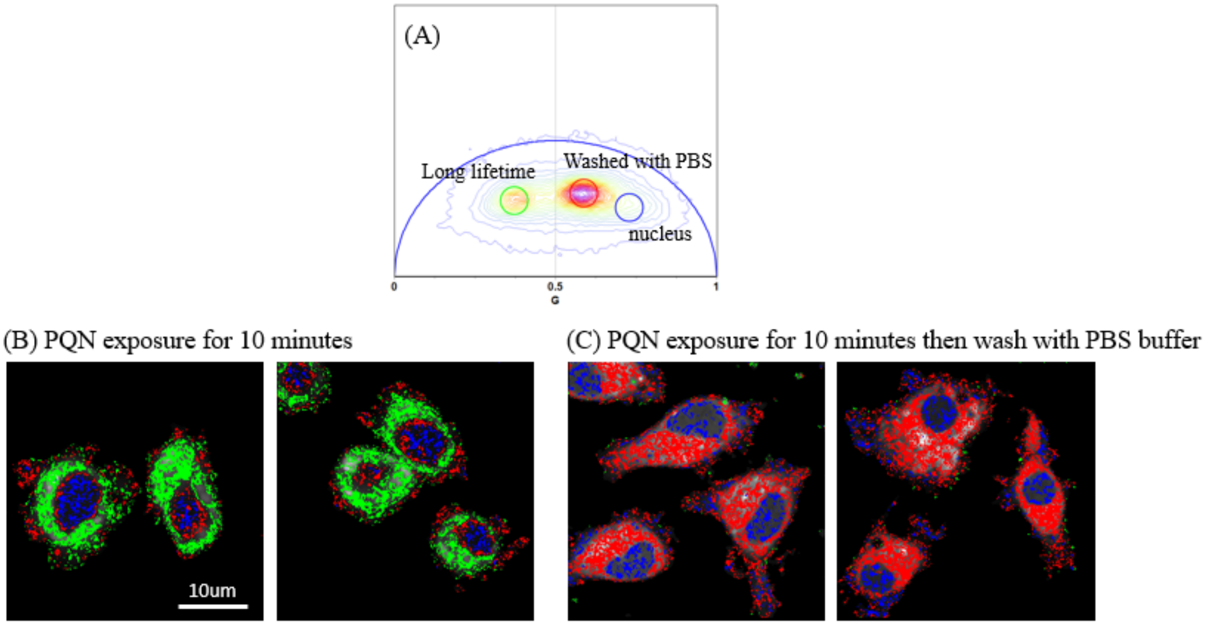

Figure S5. Phasor approach to FLIM of RAW 264.7 macrophage cells treated with 1.74 μg mL^{-1} PQN. (A) Phasor plot with blue, green, and red cursors selecting clusters with different lifetimes. (B) FLIM images of cells after 10-min exposure. (C) FLIM images of cells washed with PBS buffer after 10-min exposure.

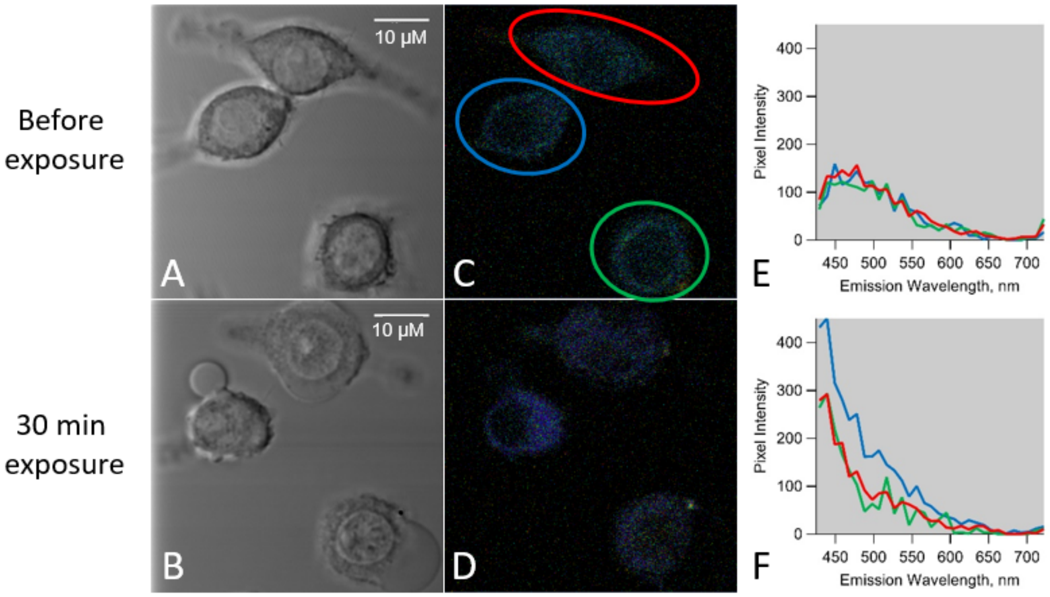

Figure S6. Hyperspectral imaging of RAW 264.7 macrophages before and after 30-min exposure to 1.74 μg mL^{-1} PQN. (A-B) are bright-field images; (C-D) are hyperspectral images, and (E-F) are emission spectra from cells highlighted with the same color of cycles (red, blue, green) in (C).

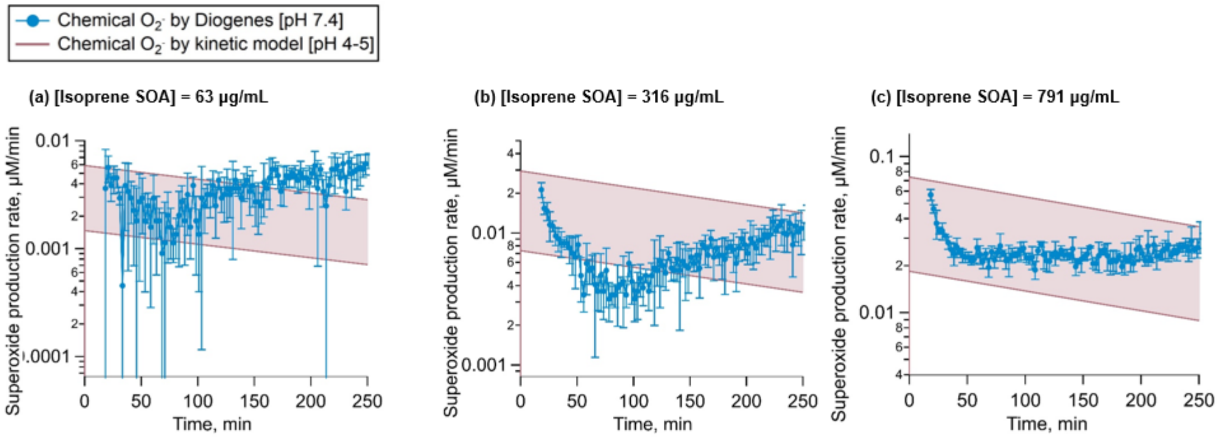

Figure S7. Chemical $\cdot O_2^-$ production rates by isoprene-derived SOA measured by the Diogenes chemiluminescence assay and model simulations using the SOA aqueous kinetic model. Data points with error bars represent the average and uncertainties calculated from error propagation based on variabilities from samples and controls. Shaded areas represent the model uncertainties associated with relative abundance of organic hydroperoxides and alcohols groups in SOA. Note that the slight increase after 50 minutes in the measurement may be due to the instability of probe under 37 ℃.

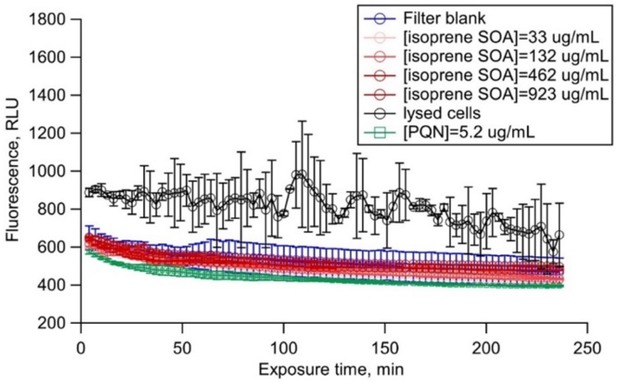

Figure S8. Time evolution of fluorescence emitted by macrophage cells exposed to PQN, extracts of filter blanks and SOA samples, and lysis solution. Experiments were performed in quadruplicate and data are presented as mean ± standard deviation.

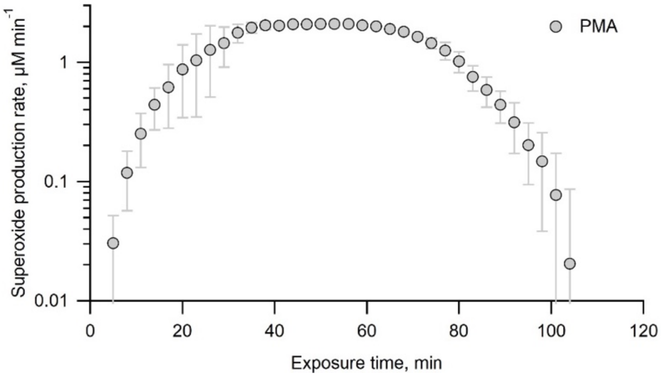

Figure S9. Time evolution of cellular $\cdot O_2^-$ production rate from macrophages stimulated with 1 μM of phorbol 12-myristate 13-acetate (PMA). Data points with error bars represent the average and uncertainties calculated from error propagation based on variabilities from samples and controls.

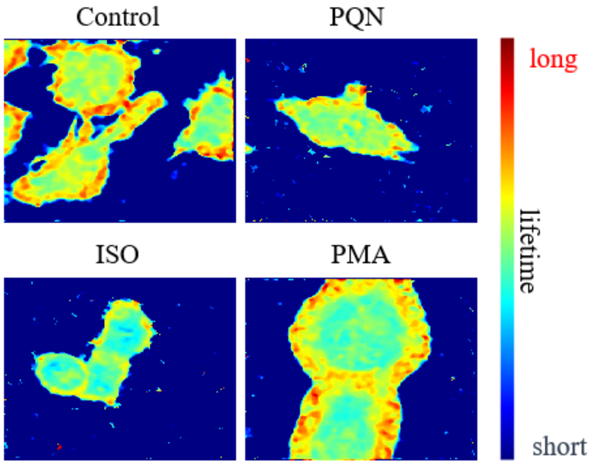

Figure S10. FLIM-Laurdan images showing the fluorescence lifetime of Laurdan dye on cell membranes of RAW 264.7 macrophage cells treated with control and various samples. Doses and exposure times are given in Table S2.

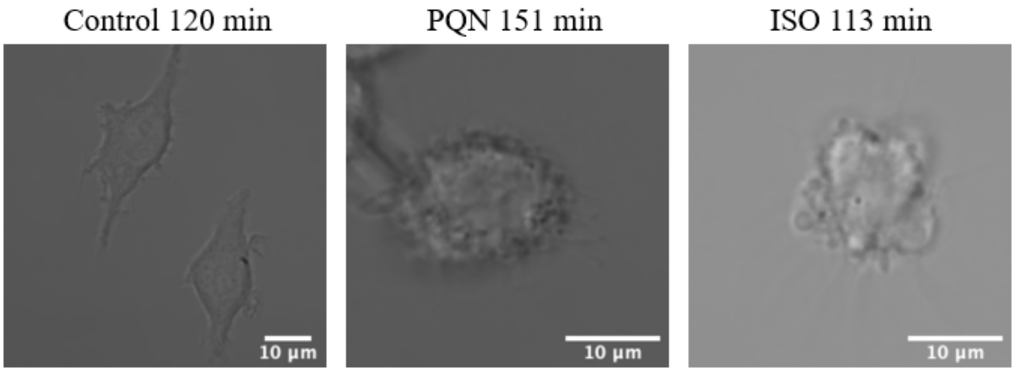

Figure S11. Bright-field cell images in FLIM showing foam cell formation after exposed to PQN and isoprene-derived SOA (ISO). Exposing times are given on top of the images.

References

1. Dikalov, S. I.; Kirilyuk, I. A.; Voinov, M.; Grigor'ev, I. A., EPR Detection of Cellular and Mitochondrial Superoxide Using Cyclic Hydroxylamines. *Free radical research* **2011,** *45*, (4), 417-430.
2. McCord, J. M., Oxygen-Derived Free Radicals in Postischemic Tissue Injury. *New England Journal of Medicine* **1985,** *312*, (3), 159-163.
3. Fridovich, I., Quantitative Aspects of the Production of Superoxide Anion Radical by Milk Xanthine Oxidase. *Journal of Biological Chemistry* **1970,** *245*, (16), 4053-4057.
4. Gotham, J.; Li, Q.; Tipple, T., 89 - Method optimization for spin probe-based electron paramagnetic resonance spectroscopic detection of oxidative species. *Free Radical Biology and Medicine* **2018,** *128*, S52.
5. Togashi, D. M.; Nicodem, D. E., Photophysical studies of 9,10-phenanthrenequinones. *Spectrochimica Acta Part A: Molecular and Biomolecular Spectroscopy* **2004,** *60*, (13), 3205-3212.
6. Golfetto, O.; Hinde, E.; Gratton, E., Laurdan fluorescence lifetime discriminates cholesterol content from changes in fluidity in living cell membranes. *Biophysical journal* **2013,** *104*, (6), 1238-1247.
7. Wan, Q.; Yang, M.; Liu, Z.; Wu, J., Atmospheric fine particulate matter exposure exacerbates atherosclerosis in apolipoprotein E knockout mice by inhibiting autophagy in macrophages via the PI3K/Akt/mTOR signaling pathway. *Ecotoxicology and Environmental Safety* **2021,** *208*, 111440.
8. Pardo, M.; Katra, I.; Schaeur, J. J.; Rudich, Y., Mitochondria-mediated oxidative stress induced by desert dust in rat alveolar macrophages. **2017,** *1*, (1), 4-16.
9. Pardo, M.; Porat, Z.; Rudich, A.; Schauer, J. J.; Rudich, Y., Repeated exposures to roadside particulate matter extracts suppresses pulmonary defense mechanisms, resulting in lipid and protein oxidative damage. *Environ Pollut* **2015,** *210*, 227-237.
10. Tuet, W. Y.; Fok, S.; Verma, V.; Tagle Rodriguez, M. S.; Grosberg, A.; Champion, J. A.; Ng, N. L., Dose-dependent intracellular reactive oxygen and nitrogen species (ROS/RNS) production from particulate matter exposure: comparison to oxidative potential and chemical composition. *Atmospheric Environment* **2016,** *144*, 335-344.
11. Wang, Y.; Puthussery, J. V.; Yu, H.; Liu, Y.; Salana, S.; Verma, V., Sources of cellular oxidative potential of water-soluble fine ambient particulate matter in the Midwestern United States. *Journal of Hazardous Materials* **2022,** *425*, 127777.
12. Zhang, Y.; Schauer, J. J.; Shafer, M. M.; Hannigan, M. P.; Dutton, S. J., Source apportionment of in vitro Reactive Oxygen Species bioassay activity from atmospheric particulate matter. *Environmental Science & Technology* **2008,** *42*, (19), 7502-7509.
13. Gali, N. K.; Yang, F.; Jiang, S. Y.; Chan, K. L.; Sun, L.; Ho, K.-f.; Ning, Z., Spatial and seasonal heterogeneity of atmospheric particles induced reactive oxygen species in urban areas and the role of water-soluble metals. *Environmental Pollution* **2015,** *198*, 86-96.
14. Nishanth, R. P.; Jyotsna, R. G.; Schlager, J. J.; Hussain, S. M.; Reddanna, P., Inflammatory responses of RAW 264.7 macrophages upon exposure to nanoparticles: Role of ROS-NFκB signaling pathway. *Nanotoxicology* **2011,** *5*, (4), 502-516.
15. Franzi, L. M.; Bratt, J. M.; Williams, K. M.; Last, J. A., Why is particulate matter produced by wildfires toxic to lung macrophages? *Toxicology and Applied Pharmacology* **2011,** *257*, (2), 182-188.
16. Xia, T.; Kovochich, M.; Liong, M.; Mädler, L.; Gilbert, B.; Shi, H.; Yeh, J. I.; Zink, J. I.; Nel, A. E., Comparison of the Mechanism of Toxicity of Zinc Oxide and Cerium Oxide Nanoparticles Based on Dissolution and Oxidative Stress Properties. *ACS Nano* **2008,** *2*, (10), 2121-2134.
17. Imrich, C.-A. W. G. A.; Ning, H. D. Y., Analysis of air pollution particulate-mediated oxidant stress in alveolar macrophages. *Journal of Toxicology and Environmental Health Part A* **1998,** *54*, (7), 529-545.
18. He, M.; Ichinose, T.; Yoshida, S.; Ito, T.; He, C.; Yoshida, Y.; Arashidani, K.; Takano, H.; Sun, G.; Shibamoto, T., PM2.5-induced lung inflammation in mice: Differences of inflammatory response in macrophages and type II alveolar cells. *Journal of Applied Toxicology* **2017,** *37*, (10), 1203-1218.

19. Daher, N.; Ning, Z.; Cho, A. K.; Shafer, M.; Schauer, J. J.; Sioutas, C., Comparison of the chemical and oxidative characteristics of particulate matter (PM) collected by different methods: filters, impactors, and biosamplers. *Aerosol Sci. Technol.* **2011,** *45*, (11), 1294-1304.
